# Mortality in Three-Toed Box Turtles (*Terrapene mexicana triunguis*) at Two Sites in Missouri

**DOI:** 10.3389/fvets.2019.00412

**Published:** 2019-11-22

**Authors:** Jamie L. Palmer, Maris Brenn-White, Stephen Blake, Sharon L. Deem

**Affiliations:** ^1^Institute for Conservation Medicine, Saint Louis Zoo, St. Louis, MO, United States; ^2^Department of Biology, Saint Louis University, St. Louis, MO, United States

**Keywords:** chelonian, urban ecology, winter kill, telemetry, survival estimate, brumation

## Abstract

Once ubiquitous, North American box turtles are experiencing reductions in abundance and range, but the magnitude of these losses is largely unknown. In Missouri, native box turtles (*Terrapene mexicana triunguis* and *Terrapene ornata ornata*) are declining across the state due to anthropogenic disturbances such as urbanization, habitat fragmentation, and vehicle collisions. Through radio-tracking over a period of 7 years, we documented the survival of adult three-toed box turtles at two sites in Missouri: Forest Park (urban park) and Tyson Research Center (TRC) (a protected rural forest). Estimated annual survival of adult turtles in Forest Park was 79% (95% CI: 0.68–0.87) while at TRC annual survival was 93% (95% CI: 0.83–0.97). The odds of annual survival for a turtle at TRC were 3.5 times that of a turtle living in Forest Park. “Winter kill,” which refers to box turtles found dead on the surface during brumation or within 2 weeks of emergence, was the most frequently documented category of mortality in Forest Park. At TRC, winter kill was not documented; however, the reasons for most deaths were unknown. These data raise questions about the potential of large urban parks as refuges for box turtles, which we may answer by future studies that compare box turtles living in multiple urban and rural settings. Our preliminary data suggest that even the largest urban parks may not be able to sustain populations of box turtles which has severe implications as urbanization continues to degrade and eliminate box turtle habitat throughout their range.

## Introduction

The Chelonia (turtles and tortoises) is the most imperiled vertebrate order, with more than half (61%) of the 356 known species threatened with extinction (1). Threats to the long-term survival of land turtles, including North American box turtles, come from anthropogenic disturbances that include habitat change (loss, fragmentation, and pollution), infectious and non-infectious disease, vehicle collisions, over-exploitation for the pet trade, and a combination of environmental impacts due to climate change (1–8). Box turtles (*Terrapene* spp.) are widespread throughout much of the United States. Most studies on box turtle ecology and health have focused on *Terrapene carolina carolina* (eastern box turtles); however two taxa that occur west of the Mississippi River, *Terrapene mexicana triunguis* [three-toed box turtle, previously *Terrapene carolina triunguis* (9)] and *Terrapene ornata ornata* (ornate box turtle), have received less attention. In Missouri, both taxa occur, and despite the state's relatively low human population density and large tracts of forest and grassland, human impacts are thought to threaten their survival (10–13). The extent of population declines for three-toed and ornate box turtles is largely unknown. Historically, the range of the three-toed box turtle in Missouri extended across the state, with the species preferring forested landscapes. Ornate box turtles favor the more open prairie habitat. Today, Missouri is a mosaic of fragmented forests and small isolated prairies intersected by highways, roads, farmland, and urban and suburban areas. The three-toed box turtle is likely in decline in Missouri due to anthropogenic change. However, it is not currently listed as a state species of conservation concern, most likely due to data deficiency (14).

The life-history strategy of box turtles involves slow maturation and reliance on high juvenile survivorship and low adult mortality to maintain stable populations (15). In long-lived turtles, high adult survival is essential to the conservation of the species (6). Turtles will limit any recovery in a population due to these life history traits. Determining survival estimates and cause of mortality at the population level are important for three-toed box turtle management and conservation given the fragmentation through most of their historic range. The only previously existing survivorship estimate for this species is over 30 years old (12) and is unlikely to reflect the current population dynamics and challenges that three-toed box turtles face today, particularly in an urban environment. Knowledge of the extent to which anthropogenic changes affect box turtle populations would be valuable for understanding the ability of these animals to respond to urbanization.

As part of a larger health and movement ecology study, we estimated annual survival rates and documented causes of mortality of radio-tagged native Missouri box turtles at two sites over a 7-year period.

## Materials and Methods

**Figure 1:**
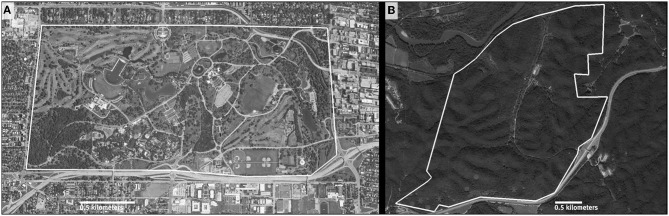
Maps of the field sites, Forest Park **(A)** and Tyson Research Center **(B)** in the mortality study of box turtles in Missouri. White line indicates site perimeter.

Since March 2012, we have monitored the movements and health of two populations of three-toed box turtles: one at an urban site and one at a rural site. Forest Park (FP), the urban site, is a 556 ha park in St. Louis, Missouri consisting of a mosaic of land use including fragmented habitats suitable for box turtles (Figure 1). Tyson Research Center (TRC), the rural site, is an 809 ha fenced and protected oak-hickory forest and biological field station (Figure 1). Research team members opportunistically located box turtles during weekly searches at both sites.

Once located, we determined sex based on morphology (16), marked each turtle with three “v” shaped notches filed into the marginal scutes (17) and placed very high frequency (VHF) telemetry tags (Holohil Systems, Ltd., Carp, Ontario, Canada) on a subset of turtles at each site (FP *n* = 23, TRC *n* = 18). We placed VHF tags only on adult box turtles considered healthy upon visual examination and weighing more than 400 g to minimize potential negative impacts of tag placement (18). The number of turtles with VHF tags varied due to turtle mortality or tag malfunction. When active, we visited each tagged turtle every 1–4 weeks to perform a visual exam, collect body weight, and record environmental data. We disinfected equipment with 10% bleach and hands with alcohol-based hand sanitizer between each turtle to minimize risk of pathogen transfer.

During brumation, usually occurring in November through March, we visited each hibernaculum every 2–4 weeks to record environmental data, disruptions in brumation, and rare instances of movement of turtles to new hibernacula. When VHF tagged turtles exhibited clinical signs, including nasal and ocular discharge, palpebral edema, lethargy, traumatic injuries, or abnormal brumation, we checked these turtles weekly to ensure retrieval of any carcasses within 1 week of death. We received wildlife collector permits through the Missouri Department of Conservation and approval from the Saint Louis Zoo Institutional Animal Care and Use Committee for this work.

Mortality data were recorded whenever a dead turtle was encountered. Cause of death was determined based on gross necropsy findings and time in relation to brumation. Five categories were used: winter kill, lawnmower, predation, non-specific trauma, or unknown. The term winter kill refers to mortality that occurs during or after brumation (15). For this study, we defined winter kill as death of a turtle found above ground during the over-wintering period or within 2 weeks of emergence from brumation and without obvious signs of trauma. Turtles found in open lawn with adjacent lawnmower tracks and/or severely fractured carapaces were classified as lawnmower mortalities. We defined predation as injury inflicted by another animal as demonstrated by trauma to limbs, head and shell with characteristics of prey interaction. Turtles with evidence of trauma that was not characteristic of either lawnmowers or predation were classified as non-specific trauma. Unknown refers to turtles found dead outside of the winter kill time period with no sign of trauma or other obvious cause of death.

We calculated an observed mortality rate as the number of confirmed deaths out of the number of total box turtles tagged. We tested for differences in observed mortality rates between sites using the Fisher exact method (fisher.exact) in R (19). To account for staggered entry of individual turtles into the study and unknown outcomes for some tagged turtles, we also estimated survival using the known-fate model, a binomial generalized linear model, in the statistical program MARK (20) and the R interface “RMark” (19, 21). As turtles were not directly observed during the months of brumation, we summarized survival data at quarterly intervals for the purposes of this analysis. To evaluate the effects of site and sex on survival in our study population, we proposed a set of four candidate models: (1) survival held constant across sites and sexes, (2) survival allowed to vary by site, (3) survival allowed to vary by sex, and (4) survival allowed to vary by both site and sex. We used Akaike's information criteria adjusted for small sample size (AICc) to compare candidate models and selected the most parsimonious model based on ΔAICc and weight. Using the selected model, we estimated annual survival probabilities and associated 95% confidence intervals. We tested for differences in survival estimates using the program CONTRAST v2.0 (22, 23).

To evaluate the different causes of mortality at each site, we calculated an observed proportionate mortality ratio for the duration of the study as the number of deaths within a specific category out of the number of deaths in all categories, including those where the cause of death was unknown. We tested for differences in proportionate mortality within and between sites using the Fisher exact method as described above.

## Results

**Figure 2:**
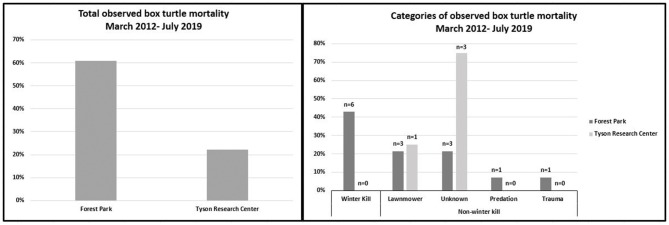
Total observed mortality in box turtles at Tyson Research Center and Forest Park. Categories of observed mortality in the mortality study of box turtles in Missouri.

Between March 2012 and February 2019, we deployed telemetry tags on 41 three-toed box turtles (FP: 11 female and 12 male; TRC: 11 female and seven male). The sex ratio of tagged turtles was comparable at each site (Fisher exact: *p* = 0.54). Over the 7 year period, the observed mortality rate in FP was 60.9% (14 out of 23) (Figure 2) consisting of eight males and six females. During this same period, the mortality rate at TRC was 22.2% (4 out of 18) (Figure 2) with three females and one male. The observed mortality rate was significantly greater at FP than TRC (Fisher exact: *p* = 0.03).

Similarly, the odds of annual survival for box turtles at TRC (93%, 95% CI: 83–97%) were 3.5 times that of box turtles in FP (79%, 95% CI: 0.68–0.87%). Of the candidate models, the model that allowed survival to vary by site was best supported with the lowest AICc, 62% of the combined model weight, and ΔAICc > 2 between it and the next best supported model (Table 1). Confidence limits for sex broadly overlapped zero in the remaining models, suggesting that sex is uninformative as a predictor of survival. We therefore used the model that allowed survival to vary only by site to predict estimated annual survival probabilities (Table 2). The model estimated coefficient for site with FP as the baseline was 1.25, which corresponds to an odds ratio of 3.49 (1.08–11.33). The difference in estimated survival probabilities between sites is statistically significant (chi-square = 5.33, *p* = 0.02).

**Table 1:** Model selection results of candidate models used to estimate survival from mortality of VHF-tagged three-toed box turtles in Forest Park (urban) and Tyson Research Center (rural) in Missouri, March 2012–February 2019.

**Model^a^**	**K**	**AICc**	**ΔAICc**	***w_***i***_***
S (site)	2	101.75	0.00	0.62
S (site + sex)	3	103.79	2.04	0.22
S (.)	1	104.76	3.01	0.13
S (sex)	2	106.74	4.99	0.05

a *Models allow survival (S) to remain constant (.) or vary by site and/or sex. We report the number of parameters estimated (K), Akaike's information criterion adjusted for small sample size (AICc), difference in AICc relative to smallest value (ΔAICc) and Akaike weights (w_i_)*.

**Table 2:** Best fit model [S (site)] estimated annual survival probabilities of VHF-tagged three-toed box turtles in Forest Park (urban) and Tyson Research Center (rural) in Missouri, March 2012–February 2019.

**Site**	**Annual survival**	**95% CI**
Forest Park	0.79	0.68–0.87
Tyson Research Center	0.93	0.83–0.97

In FP, the leading category of death was winter kill at 43%, whereas at TRC the leading category was “unknown” at 75% (Figure 2). There were no statistically significant differences in proportionate mortality between categories either within or between sites.

## Discussion

Survival estimates in our study populations are consistent with those for telemetry followed eastern box turtles (*T. c. carolina*) in Delaware and Indiana (24, 25). Specifically, the annual survival estimate at TRC (93%) is similar to those previously documented in other protected natural settings (95–98%) (24, 25), whereas FP (79%) is similar to a site with relatively high human disturbance (81%) (24).

These differences in mortality may be due to local environmental factors, including anthropogenic impacts, at both sites. TRC is a contiguous, oak-hickory forest managed by Washington University in St. Louis with one paved road and perimeter fencing that does not impede turtle movement, but does control human presence in the site. FP is bordered on one side by a highway and on three sides by city neighborhoods. The park contains a variety of habitats including fragmented forest patches, golf courses, recreational sport fields, and a number of landscaped gardens. Forest fragments are the main habitat utilized by box turtles in FP, and all are bordered by park roads and grass lawns that are regularly mowed.

Research on reptiles living in urban environments suggests that urban box turtles and other urban-dwelling reptiles are more vulnerable to mortality than less urbanized populations (24, 26–29). Threats to box turtles that may be heightened in an urban environment include collection for use in the pet trade and vehicle and lawnmower-related morbidity and mortality (24, 26). Future survival analyses conducted at multiple urban and rural sites while explicitly measuring variation in land use and habitat features would be valuable for understanding the extent to which these anthropogenic changes affect box turtle populations.

Winter kill was the most documented category of mortality in FP but was not observed for any turtles at TRC. Winter kill is thought to be caused by long periods of frigid temperatures without snow or leaf litter of sufficient depth to provide adequate insulation during brumation (15) and is not uncommon in box turtles and other turtle species (15, 30–32). Possible explanations for the observed difference in winter kill between sites include that turtles in FP have poor insulation or ability to burrow deep enough to avoid the harsh winters given the small patch size, human removal of leaf litter, and lack of canopy cover during winter months. Additionally, anthropogenic habitat disruption may adversely affect box turtle brumation. For example, many habitats in FP are managed for restoration of native vegetation and invasive species removal (honeysuckle, *Lonicera* spp. and winter creeper, *Euonymus fortunei*) or modified for human use. These practices may disrupt the microclimates of brumating turtles by removing insulating materials during key winter months. Turtles at TRC usually overwinter in the low portion of ravines under thick leaf litter and therefore may experience different microclimates during the winter than FP turtles. We are currently collecting hibernaculum and surface temperature during brumation for all tagged turtles to determine if differences in insulation efficacy exist between the two sites.

FP is also a site that we believe is popular for pet turtle drop-offs based on observations of non-native eastern box turtles and by personal communication with FP visitors. We also occasionally encounter turtles with shell deformities that are typical of captive kept box turtles (32). These released turtles might be unprepared to brumate depending on their previous husbandry; their potential inability to do so efficiently may be cause for some winter kill cases.

Box turtles are susceptible to vehicle mortality, especially females moving in search of suitable nest sites (26, 33–35). Contrary to expectation, females did not experience greater mortality due to any cause, and no road mortality occurred in tagged turtles at either site, though we did observe road mortality in non-tagged box turtles (TRC *n* = 0, FP *n* = 5). Lawnmower strikes, however, were an important cause of mortality at both sites representing 21% (*n* = 3/14) and 25% (*n* = 1/4) of proportionate mortality at FP and TRC, respectively. This demonstrates that this anthropogenic threat may affect turtles wherever human management of grasslands may occur.

Predation and non-vehicular or lawnmower trauma were only documented in FP and at low proportions at 7% (*n* = 1/14) for each. Cause of death was unknown for the majority of tagged turtles at TRC at 75% (*n* = 3/4) and a significant proportion of tagged turtles in FP with 21% of the mortality. Possible explanations for these cases include death from natural causes or diseases we could not diagnose based on gross necropsy alone depending on carcass condition. We cannot rule out the possibility of infectious disease or exposure to environmental toxins as a contributing factor or cause of death for any individual, as we did not specifically test for either at the time of death.

Limitations to our study include small sample size and inability to determine cause of death for six out of 18 turtles, thus making it difficult to interpret differences in cause-specific mortality. Testing for pathogens, toxins, and diagnostic procedures that we did not perform may help identify the cause of death in these undetermined cases and provide further data for cause of death in all the turtles. Lack of known history of these animals before tracking and the potential for some to have previously been pets may have affected data in these turtle populations that were presumed to be wild. Also, several tagged individuals at FP (*n* = 3) were lost to follow-up due to either human removal or radio-tag malfunction. Lastly, as there are no replicate urban and rural sites in our study design, associations between mortality rates and site characteristics cannot be explicitly tested and, instead, are best used for hypothesis generation for future research.

Of the identified categories of mortality, winter kill and lawnmower are both important in FP. The leading category was winter kill (43%), suggesting that successful brumation may be difficult for box turtles living in FP, but further data on habitat characteristics and quality are necessary. Because we do not know the history of these individuals, it is possible that some turtles in this study had been pets and were therefore unable to overwinter properly. Human disturbances causing habitat loss and fragmentation, however, are documented causes of population decline in box turtles (12, 24, 27, 36) and may impact the ability of turtles to successfully brumate. Environmental variation due to climate change is predicted to further intensify the rate of box turtle decline, because as ectotherms they and reliant on appropriate temperature throughout the year, including the brumation period (37). As compared to other, more long-term studies on rural Missouri box turtle survival, the higher mortality in FP box turtles may imply that urbanization may increase risk of mortality (12). While high, we do not know the impact of the observed mortality rate on box turtle population stability in FP. We recommend box turtles living in urban parks, such as FP, are monitored for population structure, reproductive success, and survival and that these data are compared to rural populations to determine whether urban parks serve as viable habitat for turtle conservation at a time of increasing urbanization throughout the U.S.

## Data Availability Statement

The datasets generated for this study are available on request to the corresponding author.

## Ethics Statement

The animal study was reviewed and approved by Saint Louis Zoo Research Animal Use Committee.

## Author Contributions

JP substantially contributed to the design of the project, field data collection, and drafting of this manuscript for publication. As the field and lab technician for this research she is involved in every aspect of the box turtle program on a day to day and program level basis. MB-W is a veterinarian for this box turtle research and provided all statistical analysis as well as contribution to the manuscript in content and edits. SB is Co-PI for the box turtle research described here and provided substantial contributions to the research design, implementation, data collection, manuscript editing, and interpretation of the work. SD is the PI for the box turtle research described here, provided substantial contributions to the research design, implementation, data collection, manuscript editing, and interpretation of the work, is the lead veterinarian for the research, and approves all research questions and implementation.

### Conflict of Interest

The authors declare that the research was conducted in the absence of any commercial or financial relationships that could be construed as a potential conflict of interest.
